# Relation between nodule size and ^18^F-FDG-PET SUV for malignant and benign pulmonary nodules. 

**DOI:** 10.1186/1756-8722-1-13

**Published:** 2008-09-22

**Authors:** Majid Khalaf, Hani Abdel-Nabi, John Baker, Yiping Shao, Dominick Lamonica, Jayakumari Gona

**Affiliations:** 1 Department of Nuclear Medicine, University at Buffalo (SUNY), Buffalo, New York, USA; 2 Department of Nuclear Medicine, Roswell Park Cancer Institute, Buffalo, New York, USA; 3 Department of Nuclear Medicine, Veteran Affairs Western New York Healthcare System, Buffalo, New York, USA; 4 PET Center, Children's Hospital of Michigan, 3901 Beaubien Blvd, Detroit, MI 48201, USA

## Abstract

The most common semiquantitative method of evaluation of pulmonary lesions using ^18^F-FDG PET is FDG standardized uptake value (SUV). An SUV cutoff of 2.5 or greater has been used to differentiate between benign and malignant nodules. The goal of our study was to investigate the correlation between the size of pulmonary nodules and the SUV for benign as well as for malignant nodules.

Retrospectively, 173 patients were selected from 420 referrals for evaluation of pulmonary lesions. All patients selected had a positive CT and PET scans and histopathology biopsy. A linear regression equation was fitted to a scatter plot of size and SUV_max _for malignant and benign nodules together. A dot diagram was created to calculate the sensitivity, specificity, and accuracy using an SUV_max _cutoff of 2.5.

The linear regression equations and (R^2^)s as well as the trendlines for malignant and benign nodules demonstrated that the slope of the regression line is greater for malignant than for benign nodules. Twenty-eight nodules of group one (≤ 1.0 cm) are plotted in a dot diagram using an SUV_max _cutoff of 2.5. The sensitivity, specificity, and accuracy were calculated to be 85%, 36% and 54% respectively. Similarly, sensitivity, specificity, and accuracy were calculated for an SUV_max _cutoff of 2.5 and found to be 91%, 47%, and 79% respectively for group 2 (1.1–2.0 cm); 94%, 23%, and 76%, respectively for group 3 (2.1–3.0 cm); and 100%, 17%, and 82%,, respectively for group 4 (> 3.0 cm). The previous results of the dot diagram indicating that the sensitivity and the accuracy of the test using an SUV_max _cutoff of 2.5 are increased with an increase in the diameter of pulmonary nodules.

The slope of the regression line is greater for malignant than for benign nodules. Although, the SUV_max _cutoff of 2.5 is a useful tool in the evaluation of large pulmonary nodules (> 1.0 cm), it has no or minimal value in the evaluation of small pulmonary nodules (≤ 1.0 cm).

## Introduction

Metabolic imaging with ^18^F-FDG PET is a well-established indication for the evaluation of pulmonary nodules. In current practice, standardized uptake value (SUV) is one of the most common methods to evaluate pulmonary nodules. Semiquantitative determination of FDG activity is obtained by calculating SUV in a given region of interest (ROI). An SUV cutoff of 2.5 or greater has been traditionally associated with malignant pulmonary nodules [1]. However, Thie (*2*) has previously reported many factors that influence the calculation of SUV. These might include: 1) the shape of ROI; 2) partial-volume and spillover effects; 3) attenuation correction; 4) reconstruction method and parameters for scanner type; 5) counts' noise bias effect; 6) time of SUV evaluation; 7) competing transport effects; and 8) body size. Factors obtained in small phantom data allow observed ROI activity to be corrected to that truly present. There is dependency on the reconstructed resolution, the size and geometry, and the ratio of activities in the ROI region and the surrounding region. Motion blurring (e.g., from the diaphragm) also undesirably averages pixel intensities [2]. In addition to the equipment and physical factors, the biological factors of the nodules have an influence on SUV. The slowly growing and well-differentiated tumors generally have lower SUVs than rapidly growing and undifferentiated ones. Bronchoalveolar and carcinoid tumors have been reported to have lower SUVs than non-small cell lung cancers [3-5]. On other hand, some acute infectious and inflammatory processes such as TB, Cryptococcus infection, and rheumatoid nodules might have high SUVs that often overlap with the SUVs of rapidly growing and undifferentiated tumors [6-8]. Moreover, different papers [9-13] reported that the semiquantitative method of SUV is not superior to the visual assessment in the characterization of pulmonary nodules, particularly for small nodules.

Despite the major role of metabolic imaging with ^18^F-FDG PET in management of pulmonary lesions, in the current clinical practice, the characterization of small pulmonary nodules remains a challenge for clinicians. The goal of our study was to investigate the correlation between the size of pulmonary nodules and the SUV for benign as well as for malignant nodules. We examined the sensitivity, specificity and accuracy of the ^18^F- FDG PET SUV_max _cutoff of 2.5 in differentiating between malignant and benign pulmonary nodules. In addition, we examined an SUV_max _cutoff of less than 2.5 for characterizing pulmonary nodules of 1.0 cm or less.

## Materials and methods

### Patients

Patients were selected retrospectively from PET center databases of Veteran Affairs Western New York Healthcare System, referred to as medical center A (MC-A) and Roswell Park Cancer Institute, referred to as medical center B (MC-B) in Buffalo, New York. Samples of 173 patients were selected from 420 referrals for ^18^F-FDG PET evaluation of pulmonary lesion(s) in the two medical centers between February 2004 and November 2005. The reminder was ineligible for the study due to unavailability of pathological diagnosis or CT-thorax; or PET scan was negative. There were 147 males and 26 females; aged 67 years ± 11.6, with a range between 25–89 years. A phantom study was performed to measure the difference in SUV between the two scanners. All patients who were selected for the study had positive CT scans of the chest for pulmonary nodule(s), a histopathology biopsy, and a positive PET scan for nodule(s) to measure the SUV. Patients who had negative PET scan, negative CT or no histopathology of the nodule(s) were excluded from the study. The last two were excluded because the SUV or the size of the nodule cannot be measured. The measurements of nodules were obtained from CT reports. All PET scans were adjusted for body weight for SUV calculation. The study was approved by Institutional review Boards (IRB) of (MC-A) and (MC-B), and given exempt status from the informed consent requirement.

### Imaging protocol of ^18^F-FDG PET scans

All patients fasted at least 4 hours before receiving a 10–15 mCi (370 MBq-555 MBq) dose of intravenous ^18^F-FDG. PET scans were performed approximately 60 minutes after the injection of the ^18^F-FDG dose. Emission and transmission acquisition times were 5 and 3 minutes, respectively, per bed position. All SUV measurements were adjusted for body weight and blood glucose was measured for all diabetic patients to ensure that it was within acceptable limits. The PET Model of MC-A Scanner was Siemens ECAT EXACT HR+ with detector type of BGO, 288 detectors (16 Crystals: 1 PNT), 18, 432 crystals (4,04 + 4.39 × 30 mm). The Axial Coverage was 15.5 cm with Spatial Resolution of TA: 5.5, A: 4.7 mm FWHM. The PET Model of MC-B Scanner was GE Advance S9110JF with detector type of BGO, 366 detectors (18) Rings, 12,096 (4 × 8 × 30 mm). The Axial Coverage is 15.2 cm with Spatial Resolution of TA: 5.5, A: 5.3 mm FWHM. Attenuation was corrected by standard transmission scanning with 68 Ge sources. Acquisition mode was 2-dimensional from skull vertex to mid thigh. Images were reconstructed in coronal, sagittal and axial tomographic planes, using a Gaussian filter with a cutoff frequency of 0.6 cycles per pixel, ordered-subset expectation maximization (OSEM) with 2 iterations and 8 subsets, and a matrix size of 128 × 128. The images were interpreted on workstations in coronal, sagittal and axial tomographic planes.

### Data and statistical analysis

Using 75% isocontour, regions of interest (ROIs) were drawn around the lesions after these were visually assessed, and identified as corresponding to the lesions on the CT scan and histopathology reports. The scanners' analysis software tools calculated both maximum and mean SUV values. After all nodules from both centers were pooled together, they were divided into 4 groups according to their longest axial dimensions. Group 1 nodules were equal or less than 1 cm in diameter; group 2 nodules ranged from 1.1-to-2.0 cm; group 3 nodules ranged from 2.1-to-3.0 cm; and group 4 nodules/mass were more than 3 cm. Nodules were separated into malignant and benign categories according to the histopathology. We thus obtained 12 groups of nodules: all nodules pooled together irrespective of pathology (n = 4), malignant nodules (n = 4) and benign nodules (n = 4). The SUV_max _with standard deviation and range, and SUV_mean _with standard deviation and range of each group were calculated using Microsoft Excel. T-tests were used to compare differences in SUV_max _values between malignant and benign nodules for the four size groups.

A linear regression equation was fitted to a scatter plot of size and SUV_max _for malignant and benign nodules together, using Microsoft Excel. A dot diagram was created using MedCalc software version 9.2 for SUV_max _cutoff of 2.5 to calculate the true positive (TP), false positive (FP), true negative (TN) and false negative (FN) rates for all nodules together and for each mixed (benign and malignant) nodule group. Accordingly, the sensitivity, specificity, and accuracy of an SUV_max _cut-off of 2.5 in differentiating between benign and malignant nodules were calculated for all nodules together and for each size group. In addition, the accuracy was calculated for all nodules of MC-A and MC-B separately. The accuracy was calculated according the following formula: Accuracy = TP+TN/TP+TN+FP+FN.

### Phantom study

A cylindrical phantom (8.5 inches diameter and 7.5 inches long) 2 sets of 5 hot spheres (from 6 to 25 mm diameters) was imaged with the scanners of MC-A and MC-B with their normal clinical protocols. One set of the spheres was concentrically located around the phantom axial line, and the other set was not, so that the location dependency of spheres would simulate the clinical cases where the nodules might be central or peripheral in the chest. Images were acquired with two target-to-background (T/B) activity ratios of FDG: 5:1 initially, and 2.5:1 with increased background activity. In order to get high quality image data, the activity concentration of the spheres at the beginning of the imaging was around 1.0 micro Ci/cc. Emission and transmission acquisition times were 5 and 3 minutes respectively. Images were reconstructed using the same software, the same methods, and the same criteria as clinical studies. ROI's were drawn to surround sphere boundaries by the investigators, and the Scanners' analysis software tools calculated both maximum and mean SUV.

## Results

### Patients characteristics

Table 1 summarizes the characteristics of patients. The populations of the two medical centers were similar in age, however, they differ in the percentage of female patients and the proportion of small nodules (≤ 1 cm). The female percentage of MC-A is very low due to the fact that the veteran patients are predominantly male. The proportion of small nodules for MC-A was 9% and for MC-B was 23%. The difference in the proportion of small nodules between the two centers may be related to differences in the protocols of the two medical centers to evaluate and follow up small pulmonary nodules.

**Table 1:** Characteristics of patients

Variable	MC A	MC B	Total
No. of patients	110	63	173
Mean age (Range)	68 (46–89)	66 (25–89)	67 (25–89)
Male (5%)	108 (98)	42 (67)	150 (87)
Female (%)	2 (2)	21 (33)	23 (13)
All Nodule	127	75	202
Malignant (%)	92 (72)	55 (73)	147 (72)
Benign (%)	35 (28)	20 (27)	55 (28)
Nodules ≤ 1 cm	11	17	28
Malignant (%)	4 (37)	9 (53)	13 (47)
Benign (%)	7 (63)	8 (47)	15 (53)

MC = medical center

### Characteristics of nodules

**Figure 1:**
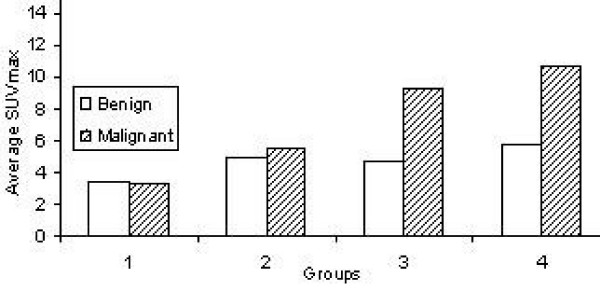
Histogram of malignant versus benign nodules for groups one to four.

Table 2 summarizes the characteristics of nodules. One of the main findings in table 2 is that the percentage of malignancy increases as the nodule size increases. It increased from 47% for group 1 to 80% for group 4. Another significant finding is the average SUV_max _of benign nodules increased from 3.34 for small nodule (≤ 1 cm) to 5.78 for nodules/mass (> 3 cm), while average SUV_max _of malignant nodules increased from 3.28 for small malignant nodules to 10.67 for large malignant nodules (Figure 1). The increase in the average SUV_max _was more prominent for malignant nodules than benign nodules indicating that there is a stronger relation between the SUV_max _and the size of the malignant nodule groups than for benign nodules. The histopathology of malignant and benign nodules is listed in table 3.

**Table 2:** Characteristics of nodules

		Number of nodules			SUV_max_	
			
Groups	MS in cm	Total	M (%)	B (%)	M (SD)	B (SD)
≤ 1.0 cm	0.78	28	13 (47)	15 (53)	3.28 (1.28)	3.34 (1.09)
1.1–2.0 cm	1.58	58	42 (72)	16 (28)	5.52 (2.64)	4.90 (3.98)
2.1–3.3 cm	2.61	47	36 (76)	11 (24)	9.27 (5.33)	4.67 (2.72)
> 3.0 cm	5.08	69	55 (80)	14 (20)	10.67 (4.84)	5.78 (3.12)

MS = mean size, cm = centimeter, M = malignant, B = benign, SD = standard deviation

**Table 3:** Histopathology of malignant and benign nodules

HP of malignant nodules (*n *= 147)	Number of nodules (%)
Adenocarcinoma	59 (40)
Squamous cell carcinoma	40 (27)
Large cell cancer	11 (7.5)
Carcinoid tumor	11 (7.5)
Non-specified NSCLC	9 (6.1)
Small cell lung cancer	8 (5.4)
Other	9 (6.1)
HP of benign nodules (*n *= 55)	

Non-specified benign	10 (18)
Fibrosis-elastosis	9 (16)
Chronic inflammation	7 (13)
Lymphoid tissue hyperplasia	4 (7.2)
Squamous metaplasia 4	4 (7.2)
Granuloma	3 (5.5)
Atypical cytology	3 (5.5)
Tuberculosis	3 (5.5)
Rheumatoid nodules	2 (3.6)
Silicoanthracotic nodules	2 (3.6)
Cryptococcus infection	2 (3.6)
Other	6 (11)

HP = Histopathology

### Result of the phantom study

Spheres with diameters 10 to 25 mm were confidently identified in all images for 5:1 T/B ratio, and 16 to 25 mm for 2.5:1 ratio. The data has shown that SUV values from two different scanners follow a very similar function with respect to the sphere sizes, and the values from the scanner of MC-A were consistently ~1.3× higher than the ones from the scanner of MC-B.

### Data analysis-linear regression equation

**Figure 2:**
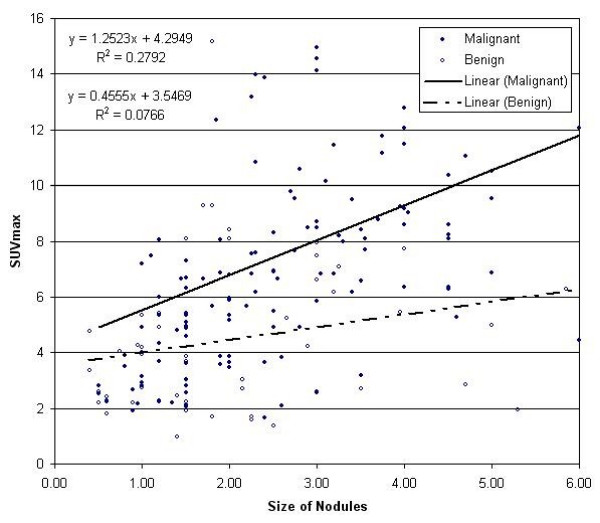
Linear regression equation fitted to all malignant and benign nodules.

A linear regression equation fitted to all malignant and benign nodules was generated using Microsoft Excel spreadsheet. For malignant nodules, the linear regression equation parameters and percentage of variance accounted for (R^2^) were (y = 1.2523x + 4.2949) and (R^2 ^= 0.2492). The linear regression equation parameters and (R^2^) for benign nodules were (y = 0.4555x + 3.5469) and (R^2 ^= 0.0766). The equations and trendlines demonstrate that the slope of the regression line is greater for malignant than for benign nodules. The larger the diameter of the malignant nodule is, the higher the possibility of a higher SUV. As the pathology of malignant nodules distributed randomly, the smaller nodules tended to have lower SUV than larger nodules of the same pathology (Figure 2).

Statistical analysis using t-tests revealed that there were no significant differences in SUV_max _values between malignant and benign nodules for Group 1 (t (26) = 0.3, ns) and for Group 2 (t (56) = -0.2, ns). The differences in SUV_max _values between malignant and benign nodules did reach statistical significance for Group 3 (t (44) = -3.1, p < .004) and for Group 4 (t (65) = -3.3, P < .002).

Accordingly, SUV_max _becomes useful as a tool to differentiate between malignant and benign lesions for larger nodules. However, when we examine the standard deviation (SD) of the average of the SUV_max _for larger malignant and benign nodules, there is obvious overlap. There was no predetermined fixed SUV cutoff that able to differentiate pulmonary nodules as definitely benign or definitely malignant, regardless of the nodule size (Table 2).

### Data Analysis-dot diagram

**Figure 3:**
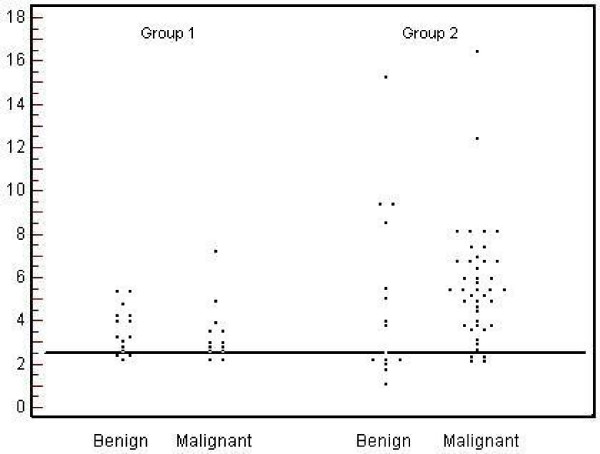
Dot diagram for groups one and two using SUV_max _cut-off of 2.5.

A total of two hundred-and-two nodules of all groups were plotted in a dot diagram, using an SUV_max _cutoff of 2.5. The number of TP, FP, TN and FN nodules was 138, 40, 15 and 9, respectively. The sensitivity, specificity, and accuracy were calculated to be 93%, 27% and 76%, respectively. Since all negative PET scan were excluded from the study, the sensitivity, specificity, and accuracy mentioned in this study do not apply for PET as a test but for SUV_max _cutoff of 2.5 as a test. Twenty-eight nodules of group 1 were plotted in the same manner. The sensitivity, specificity, and accuracy was 85%, 36% and 54% respectively (Figure 3), compared to 91%, 47%, and 79% for nodules in Group 2 (1.1 – 2.0 cm). These values tended to improve with increasing size of nodules. Using a SUV_max _cutoff of 1.8 or less for the smaller nodules increased the sensitivity to 100% from 85%; however, there were decline in the specificity and the accuracy of the test to differentiate between the malignant and benign nodules.

## Discussion

The data of this study is collected from two PET centers, a phantom study is used to examine the SUV measurement on both scanners. The experiment indicates that SUV from different scanners under the same image protocols and same scintillation detector type (BGO for both scanners) can be quite different in value. However, they follow very similar trends as size increases, the SUV value increased despite all spheres having the same T/B activity ratios, which is consistent with our clinical result. Accordingly, we recommend that the follow up scans to evaluate treatment response or re-stage the disease be performed on the same scanner to be comparable. The difference in SUV on different scanners despite the same T/B activity ratios might be attributed to the difference in calibration and machine-identity-features. Although, there was a difference in the SUV_max _value between our two scanners of a factor of ~1.3× in the phantom study, we chose not to apply an adjustment of SUV_max _for our clinical result because the average SUV_max _of each nodule group from both centers were close to each other, particularly for group 1 and group 2. The averages of the SUV_max _of group 1 and group were 3.03 and 5.28 for MC-1, respectively, and 3.3 and 5.43 for MC-2, respectively. In addition, overall accuracy using an SUV_max _cutoff of 2.5 were similar. The accuracies were 77% and 75% for MC-1 and MC-2, respectively. The trendline, linear regression equation and R^2 ^of malignant and benign nodules for MC-1 and for MC-2 demonstrate the same relation between nodule size and SUV_max_. The relation is stronger for malignant than benign lesions. Consequently, we selected to keep the clinical data as it is without adjustment of SUV_max _between the two scanners.

The results of the present study indicate that there is a relation between the size of pulmonary nodules and the SUV value. The linear regression equation and R^2 ^for malignant nodules and for benign nodules, as well as the trendlines for malignant and benign nodules demonstrated that the slope of the regression line was greater for malignant than for benign nodules. In Figure 2, it can be seen that on the left side of the graph, where the small nodules (≤ 1 cm) are plotted, the nodules mixed randomly with no predominant areas for benign or malignant nodules. No SUV_max _cutoff can separate them. However on the middle and right side, where larger size nodules (> 2.0 cm) are plotted, the nodules become more polarized, and the malignant nodules predominate in the upper portion of the plot area where the SUV is high, while the benign nodules predominate in the lower portion of the plot area where SUV is lower. Determination of an SUV cutoff for larger nodules is more feasible but not definite in the diagnosis of pulmonary nodules.

When the SUV_max _cutoff of 2.5 was used to differentiate between malignant and benign pulmonary nodules. The sensitivity, specificity and accuracy of nodules for group 2 was 91%, 47%, and 79%, respectively. For group 3 it was 94%, 23%, and 76%, respectively. For group 4 it was 100%, 17%, and 82%, respectively. Although, the sensitivity and accuracy of the test increased with the increase in the size, reaching 100% and 82% respectively for nodules greater than 3.0 cm, the specificity declined from 47% for group 2 to 17% for group 4. The accuracy of differentiating large pulmonary nodules (> 1.0 cm) using SUV_max _cutoff of 2.5 seems reasonable. However, no predetermined fixed SUV_max _cutoff is able to differentiate pulmonary nodules as definitely benign or definitely malignant, regardless of the nodule's size.

One of the main findings of the present study was that the small nodules (≤ 1 cm) tend to have lower SUVs than larger nodules. The small benign pulmonary nodules have average SUV as equal as to malignant nodules. Thus, maximum or mean SUV is not accurate tool in the evaluation of small pulmonary nodules. Only 54% of the time was the test able to differentiate between malignant and benign nodules. Attempting to lower SUV_max _to less that 2.5, such as 1.8 might increase the sensitivity of the test, however, the specificity is decreased resulting in no clinically significant improvement in the accuracy of the test to differentiate between the malignant and benign nodules. The sensitivity, specificity, and accuracy of a cutoff of 1.8 were 100%, 0.0%, and 46%, respectively. This result reflects the fact that FDG is not a specific tracer for malignancy. In our study, a variety of small benign nodules (≤ 1 cm) presented with mean and maximum SUV more than 2.5 and resulted in a false positive PET scan. (e.g., the SUV_max _was 5.3 for squamous metaplasia, 4.6 for rheumatoid nodules, 4.2 for lymphoid tissue and 3.9 for TB). Other benign nodules such as granuloma, chronic inflammation, cryptococcus infection, reactive nodules and atypical hyperplasia also presented with high SUV_max _leading to reading a false positive PET scan. On the other hand, some of well-differentiated and slow growing malignant nodules presented with SUV_max _less than 2.5 (1.34 for squamous cell carcinoma, 1.77 for adenocarcinoma and 2.15 for small cell lung cancer).

The data above support that although, the SUV_max _cutoff of 2.5 is a useful tool in the evaluation of large pulmonary nodules (> 1.0 cm), it has no or minimal value in the evaluation of small pulmonary nodules (≤ 1.0 cm). However, the combination of flexible value of SUV_max _cutoff according to the size of the nodule, visual assessment, and CT characteristics of the nodules, in addition to pretest probability of malignancy, is the most appropriate approach to characterize small pulmonary nodules. To increase the sensitivity of the test of SUV_max _cutoff for characterizing small nodules (≤ 1 cm), we recommend reducing the cutoff of less than 2.5

The limitation of this study is the exclusion of the negative PET scans. We exclude negative PET scan because the SUV of a non-FDG-avid nodule cannot be measured. Thus, the specificity of PET scan using an SUV_max _cutoff of 2.5 calculated on this study is not reflecting the actual specificity of PET in the characterizing of pulmonary nodules

The introduction of dedicated PET/CT scanners to the clinical arena in early 2001 [14], has resulted in improved accuracy in the characterization of pulmonary nodules [13], by maintaining the synergism between the anatomic sensitivity of CT, and metabolic specificity of PET.

Although, FDG-PET/CT is a valuable diagnostic tool, it has multiple pitfalls that limit its accuracy in the evaluation of pulmonary nodules, particularly small nodules. There are three potential directions for future research to improve PET/CT accuracy in the evaluation of pulmonary nodules. One direction involves improvement of PET/CT scanner to provide better sensitivity, resolution and co-registration which potentially enhance its sensitivity to detect small pulmonary nodules, in addition to provide better quantitative and qualitative evaluation of pulmonary nodules. The second direction of future research involves imaging processing and display formats that might enhance the reader delectability. A PET/CT with virtual bronchoscopy provides virtual 3-dimensional images which enhances the intraluminal lesions [15]. The third direction involves development and investigation of new PET radiotracers that might have better sensitivity and specificity to differentiate pulmonary nodules. Both ^18^F-fluorothymidine (^18^F-FLT) and ^18^F-fluorocholine (^18^F-FCH) have been developed and investigated for use in lung cancer [16-18], however neither tracer has shown clear improvement over ^18^F-FDG. Eventually, these three directions of future research will improve the delectability and categorization of the pulmonary nodules.

## Conclusion

The slope of the regression line is greater for malignant than for benign nodules. Although, the SUV_max _cutoff of 2.5 is a useful tool in the evaluation of large pulmonary nodules (> 1.0 cm), it has no or minimal value in the evaluation of small pulmonary nodules (≤ 1.0 cm).

## Competing interests

The authors declare that they have no competing interests.

## Authors' contributions

MK curried out the collection of the data, design of the study, data analysis and drafting of the manuscript. HN conceived of the study; participated in design of the study and the draft of the manuscript. JB curried out the statistical analysis; participated in design of the study and the drafting of the manuscript. YS curried out the phantom study. DL participated in the data analysis and study coordination. JK participated in the data analysis and study coordination.
